# Reproduction of Tissue by Sponge Grafting

**Published:** 1885-09

**Authors:** W. H. Atkinson


### REPRODUCTION OF TISSUE BY SPONGE GRAFTING.

BY W. H. ATKINSON, M. D., D. D. S.

READ BEFORE THE DENTAL SOCIETY OF THE STATE OF NEW YORK, AT ITS SEVENTEENTH ANNUAL MEETING AT ALBANY, MAY, 1885.

In the whole history of surgery there is no method of so much value and so little understood and practiced as that which has been called sponge grafting. Like most of the knowledge of principles and modes of practice in our specialty, it grew out of the misfortunes of traumatic or functional lesion.

The first use made of sponge grafting, in a crude way, was by obstetricians to fill the gap of cervical fissure of the uterus, resulting from violent and tedious labor.

I remember the discussions as to what became of the sponge. In one case of a very nervous woman, then called "hysterical," it was questioned whether she did not purposely remove the sponge, although she stoutly maintained the contrary. The cases submitted to this treatment did so well that this method was kept secret among a few for years. At that time, a solution of bichloride of mercury in water, alone or with sal ammoniac, was the external remedy used for scabies (itch).

Dr. William Woodruff, of Meadville, Pa., at the suggestion of a pupil then studying with him, wrung out a sponge in this solution, and used it in a badly torn cervix uteri following labor, which healed so kindly as to induce him to adopt it in the general treatment of these lesions.

The tediousness of the drainage method of healing large loss of tissue by fibrous tents, rubber tubes, or horse hair or other setons, to induce granulation, is greatly abridged by the use of the sponge graft in place of the tent, as the latter must be removed before the wound can be completely closed by the new growth. Efforts to assist nature in healing wounds and in reproduction of lost tissues from traumatic lesion or from functional lesion had been so various, bungling and inhuman, that it is no wonder that a mere expectant course long held dominion in the annals of surgery. From the abhorrent use in surgery of the actual cautery in the shape of boiling oil, into which the bleeding stump was plunged, red-hot irons and embers applied to bleeding wounds, and the potential cautery in the shape of the strongest alkalies and acids to arrest hemorrhage, the wait-and-watch treatment was established. The lack of any just apprehension of the process of inflammation permitted the introduction of "irrigation" and "drainage" of wounds and abscesses as helps to "granulation," as healing of suppurating tissue was then called.

Healing with first intention has always been regarded with high favor. Every method and management conducive to this form of healing must be of the highest importance. Transplanting bits of skin and epithelial tissue led to such kindly results as to suggest the introduction of some vehicle into which the blood plasm might be received and held in place, to form a clot in the gap of the wound, to be metamorphosed into the scar tissue, taking the place of the lost substance. Many forms of animal sutures and of flesh tents were resorted to, especially fibrous tissue, in the shape of tendons.

At length sterilized tents were introduced, which were gradually extruded as the new formations progressed in the depths of the wound, and had to be cut off from time to time, until at last a mere shred remained in the pit left at the site of the former wound tract. Sterilization is now known as "Listerism," and is a great step in advance of the old filthy method of uncleanly carelessness.

The accounts of chicken's flesh and other forms of non-human animal flesh being used to fill gapes of lost tissue, may be dismissed with the verdict of "not proven."

The accounts of the use of sterilized sponge on the other side of the Atlantic led me to try the method in my own practice. The results have been so favorable as to induce me to write this paper, with the hope of having my brethren become partakers of the benefits of this simple and beneficent treatment. Take a case where a portion of the flesh has been quite removed by the bite of a dog, the cut of a knife, buzz-saw, or other free cutting instrument. It is only necessary to stanch the bleeding, and fit a bit of sterilized sponge of the size and shape of the lost flesh, and to cover it with some impervious dressing of oiled silk, sheet rubber, court plaster, gold beater's skin, husband's plaster, or such like material, over which a light support without pressure should be secured, to keep the exudate of blood pressure from escaping too freely.

When this is done in a healthy subject, we may look for union by first intention, without one drop of pus or deteriorated product of the albuminoid clot which fills the cavity, and out of which the new growth of tissue comes. Destruction of all disease germs which adhere to the dressings constitutes the sterilization, or "Listerism," which proves so conducive to healing by first intention.

A large list of germicides is at our command, which are competent to effect sterilization. The chief one, and leading the list, is a solution of bichloride of mercury, of from one in five hundred to one in a thousand parts of water.

Sponge grafts conserve the time and energy of the healing process. The former manner of determining the point at which to cease dressing was to place the finger upon the new growth, and if the plasm broke short without adhering to the finger and forming a more or less tenacious rope, which broke as the finger was carried away farther and farther, it was deemed necessary to continue stimulating dressings; but if the rugæ of the finger left their imprint upon the jelly-like mass, closing the wound, it was deemed better to protect it from outward disturbance by a simple non-irritative dressing. If the plasm were so watery as not to rope at all, dressing with coagulant was advisable.

With the old training to guide us, and the present better understanding of the character and styles of the inflammatory process, we are more able to discriminate the indications of favorable progress and unfavorable progress of the healing process.

